# Impaired NEPHRIN localization in kidney organoids derived from nephrotic patient iPS cells

**DOI:** 10.1038/s41598-021-83501-9

**Published:** 2021-02-17

**Authors:** Tomoko Ohmori, Shankhajit De, Shunsuke Tanigawa, Koichiro Miike, Mazharul Islam, Minami Soga, Takumi Era, Shinichi Shiona, Koichi Nakanishi, Hitoshi Nakazato, Ryuichi Nishinakamura

**Affiliations:** 1 Department of Kidney Development, Institute of Molecular Embryology and Genetics, Kumamoto University, 2-2-1 Honjo, Chuo-ku, Kumamoto, 860-0811 Japan; 2 Department of Cell Modulation, Institute of Molecular Embryology and Genetics, Kumamoto University, Kumamoto, 860-0811 Japan; 3 Department of Pediatrics, Oita Prefectural Hospital, Oita, 870-0855 Japan; 4 Department of Child Health and Welfare (Pediatrics), Graduate School of Medicine, University of the Ryukyus, Okinawa, 903-0215 Japan; 5 Genetic Consulting Unit, University of the Ryukyus Hospital, Okinawa, 903-0215 Japan; 6 Department of Pediatrics, Faculty of Life Sciences, Kumamoto University, Kumamoto, 860-8556 Japan

**Keywords:** Developmental biology, Stem cells, Diseases, Nephrology

## Abstract

Mutations in the *NPHS1* gene, which encodes NEPHRIN, cause congenital nephrotic syndrome, resulting from impaired slit diaphragm (SD) formation in glomerular podocytes. We previously reported NEPHRIN and SD abnormalities in the podocytes of kidney organoids generated from patient-derived induced pluripotent stem cells (iPSCs) with an *NPHS1* missense mutation (E725D). However, the mechanisms underlying the disease may vary depending on the mutations involved, and thus generation of iPSCs from multiple patients is warranted. Here we established iPSCs from two additional patients with different *NPHS1* mutations and examined the podocyte abnormalities in kidney organoids derived from these cells. One patient had truncating mutations, and NEPHRIN was undetectable in the resulting organoids. The other patient had a missense mutation (R460Q), and the mutant NEPHRIN in the organoids failed to accumulate on the podocyte surface to form SD precursors. However, the same mutant protein behaved normally when overexpressed in heterologous cells, suggesting that NEPHRIN localization is cell context-dependent. The localization of another SD-associated protein, PODOCIN, was impaired in both types of mutant organoids in a cell domain-specific manner. Thus, the new iPSC lines and resultant kidney organoids will be useful resources for dissecting the disease mechanisms, as well as for drug development for therapies.

## Introduction

The glomerulus is the filtration apparatus within the kidney, and the podocytes in glomeruli are essential for the filtration process^1,2^. Many cytoplasmic protrusions of podocytes, termed foot processes, interdigitate with those of adjacent podocytes. The slit diaphragm (SD) located between these foot processes serves as a filtration sieve that prevents high-molecular-weight serum proteins from leaking into the urine^3,4^. The SD comprises NEPHRIN (encoded by *NPHS1*) and PODOCIN (encoded by *NHPS2*), the latter of which binds to the cytoplasmic region of NEPHRIN and stabilizes the SD.

*NPHS1* mutations cause an autosomal recessive disorder called Finnish-type congenital nephrotic syndrome, with most affected patients exhibiting severe proteinuria soon after birth^5^. The *NPHS1* gene consists of 29 exons and encodes NEPHRIN, a transmembrane protein that contains eight immunoglobulin (Ig)-like domains and a fibronectin-like domain in its extracellular region. Many mutations in *NPHS1* have been reported^6–8^, among which truncating mutations result in absence of NEPHRIN protein and loss of the SD^9,10^. For analysis of point mutations associated with amino acid substitutions, the mutant NEPHRIN proteins are often overexpressed in heterologous cell lines^7,11^. Many of these mutant proteins with amino acid substitutions fail to localize on the cell surface^11^, and thus the related point mutations probably affect protein folding, leading to retention and degradation of the misfolded proteins in the endoplasmic reticulum (endoplasmic reticulum-associated degradation: ERAD)^12^. However, some overexpressed NEPHRIN mutant proteins with amino acid substitutions show successful localization on the cell surface^11^, suggesting the existence of other mechanisms than protein misfolding. Alternatively, heterologous cell lines may be unsuitable to analyze the effects of these mutations, because the cells lack SD-associated proteins or SD structures. Although immortalized podocyte cell lines are also frequently used, their expression levels of SD-associated proteins remain low and no SD structures are detected^13–15^. These features of conventional cell lines may partly reflect their two-dimensional culture settings.

We previously reported the generation of three-dimensional kidney organoids from human induced pluripotent stem cells (iPSCs)^16^. We then applied our multistep protocol to patient-derived iPSCs. Briefly, we established iPSCs from a patient (Patient 0) with an *NPHS1* missense mutation in the spacer region (E725D) that is located between the 6th and 7th Ig-like domains, and generated kidney organoids^17^. In the resulting kidney organoids, the mutant NEPHRIN failed to localize on the cell surface of the induced podocytes, and SD precursors, which are normally formed on the basolateral domains of podocytes, were minimally detectable. Thus, the kidney organoids from the patient-derived iPSCs reproduced NEPHRIN and SD abnormalities during the initial phase of congenital nephrotic disease^17^. These organoids can be useful for screening of potential therapeutic compounds. However, the mechanisms underlying the disease, as well as the responsiveness to therapeutic compounds, may vary depending on the types of mutations involved, and thus generation of iPSCs from multiple patients is warranted. In the present study, we identified two additional patients with different *NPHS1* mutations. One patient had truncating mutations, and the other patient had a missense mutation (R460Q), for which the mutant NEPHRIN was reported to localize on the cell surface following overexpression in heterologous cells^7^. We established iPSCs from these patients and examined the podocyte abnormalities in kidney organoids derived from these cells.

## Results

### Identification of *NPHS1* mutations in two nephrotic patients

**Figure 1:**
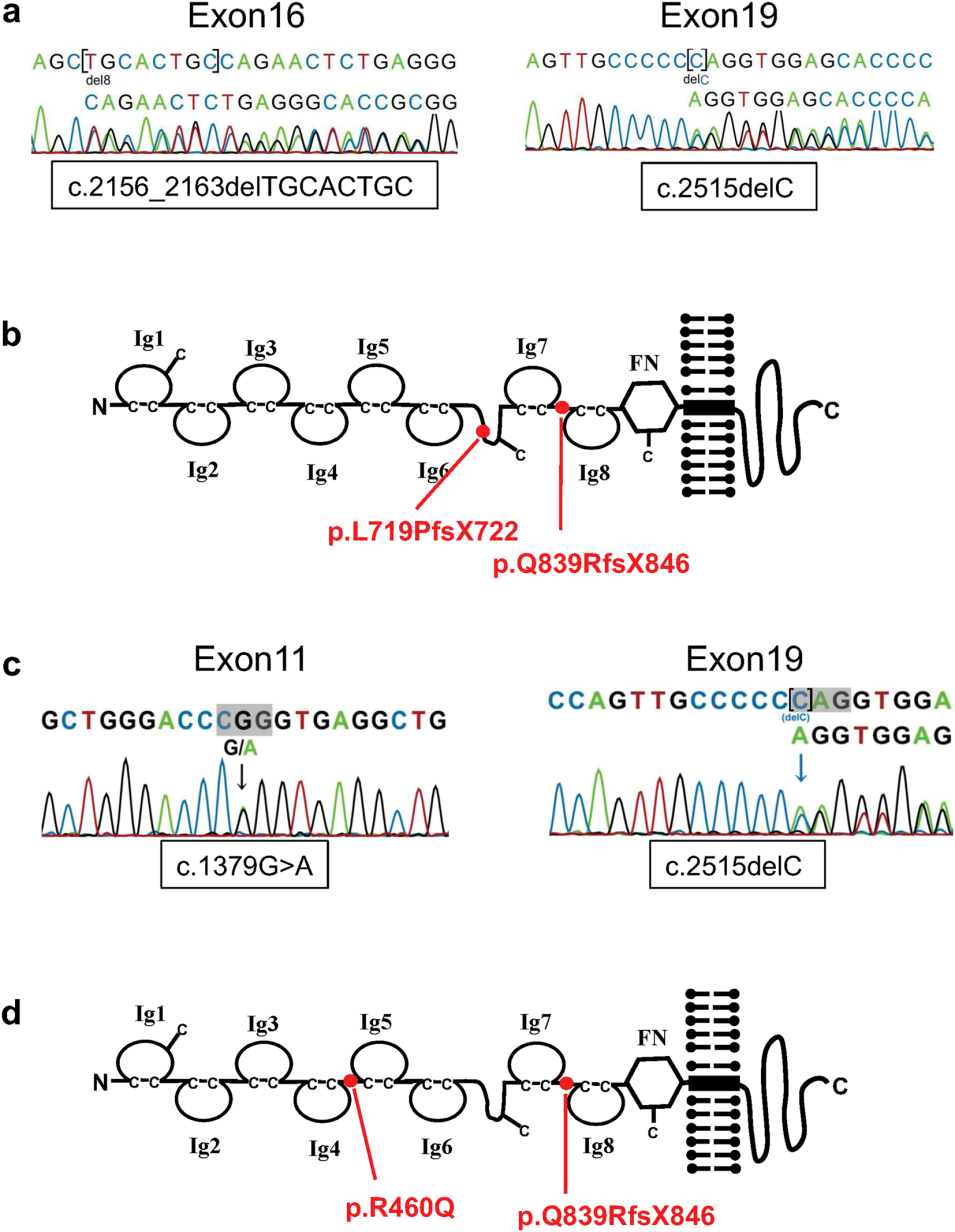
Identification of *NPHS1* mutations in two nephrotic patients. (**a**,**b**) Mutations in the *NPHS1* gene and NEPHRIN protein in Patient 1 (biallelic frameshift mutations). (**c**,**d**) Mutations in the *NPHS1* gene and NEPHRIN protein in Patient 3 (missense mutation and frameshift mutation). Ig: immunoglobulin-like domain; FN: fibronectin-like domain. The mutations on the two alleles are shown in one scheme.

Among the recruited patients with congenital nephrotic syndrome caused by *NPHS1* mutations, we focused on two patients in this study. Patient 1 and Patient 3 both exhibited severe proteinuria, edema, and reduced serum albumin level soon after birth. While Patient 1 had no family history, the sibling of Patient 3 suffered from congenital nephrotic syndrome and died within 1 month after birth. In Patient 1 (male), we identified mutations in two exons of *NPHS1*: an 8-bp deletion in exon 16 (c.2156_2163delTGCACTGC) and a 1-bp deletion in exon 19 (c.2515delC), resulting in truncation of the extracellular regions of NEPHRIN (p.L719PfsX722 and p.Q839RfsX846, respectively) (Fig. 1a,b). These mutations were located in different exons from the typical truncating mutations Fin_major_ and Fin_minor_ that occur in exons 2 and 26, respectively^5^. Although the *NPHS1* genes in his parents were not sequenced, Patient 1 likely possessed compound heterozygous mutations that resulted in non-functional alleles. Patient 3 (male) had a point mutation in exon 11 (G1379A) that resulted in an amino acid substitution (R460Q), in addition to a truncating mutation in exon 19 (c.2515delC, p.Q839RfsX846) that was identical to one of the mutations in Patient 1 (Fig. 1c,d). Sequencing of the *NPHS1* genes in his parents revealed that the missense point mutation was maternally derived, while the truncating mutation was paternally derived. Thus, the point mutation (R460Q) between the 4th and 5th Ig-like domains, combined with the non-functional truncated allele, was the likely cause of his nephrotic disease. Indeed, the same missense mutation (R460Q) was previously reported in Finnish-type congenital nephrotic syndrome^6–8^, as well as in focal segmental glomerulosclerosis^18^.

### Missense mutation does not impair NEPHRIN expression in heterologous cells

**Figure 2:**
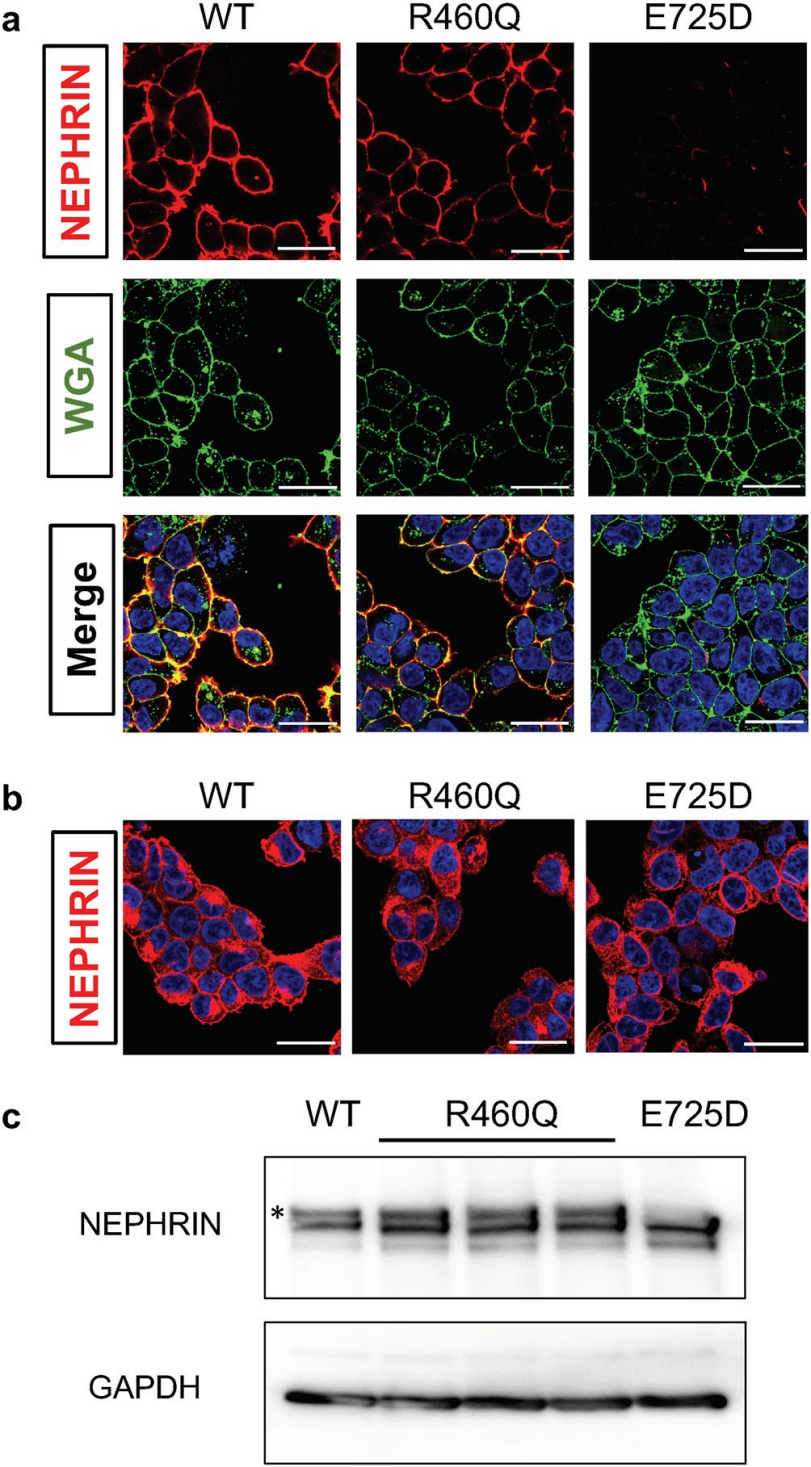
R460Q NEPHRIN mutant protein is localized on the surface in HEK293 cells. (**a**) Immunostaining of HEK293 cells overexpressing wild-type (WT), R460Q mutant (Patient 3), and E725D mutant (Patient 0) NEPHRIN proteins, using an antibody against the extracellular domain of NEPHRIN in the absence of detergent. Wheat germ agglutinin (WGA) binds to the surface glycoproteins. Scale bars, 25 µm. (**b**) Immunostaining of HEK293 cells overexpressing wild-type (WT), R460Q mutant (Patient 3), and E725D mutant (Patient 0) NEPHRIN proteins, using an antibody against the intracellular domain of NEPHRIN in the presence of detergent. Note the comparable expression of the WT and mutant NEPHRIN proteins. Scale bars, 25 µm. (**c**) Western blotting analysis of HEK293 cells overexpressing wild-type (WT), R460Q mutant (Patient 3), and E725D mutant (Patient 0) NEPHRIN. The upper band of NEPHRIN (*) is retained in the R460Q mutant, but is absent in the E725D mutant. Three R460Q mutant clones were analyzed to exclude clonal variability. The result for the E725D mutant is consistent with our previous report^17^. The results from the different parts of the same blot were combined, as depicted by the solid boxes.

A previous study utilizing immunostaining showed that NEPHRIN with the R460Q mutation successfully localized on the cell surface when overexpressed in the heterologous HeLa cell line^7^. To confirm this observation, we overexpressed wild-type and mutant (R460Q) NEPHRIN in HEK293 cells. We employed site-directed stable integration to minimize clonal variations in expression levels, and compared the results with those of the mutant (E725D) NEPHRIN from Patient 0 that fails to localize on the cell surface^17^. Immunostaining in the membrane non-permeable condition showed that both the wild-type and R460Q mutant proteins localized to the cell surface, while the E725D mutant protein did not (Fig. 2a). Meanwhile, immunostaining in the membrane permeable condition revealed comparable expression inside the cells for all three types of NEPHRIN proteins (Fig. 2b). Western blotting analysis detected two bands for wild-type and R460Q mutant NEPHRIN, while the upper band was missing for the E725D mutant NEPHRIN (Fig. 2c, Supplementary Fig. S1). Because previous studies by ourselves and others involving biotin-mediated labeling of cell surface proteins showed that the upper band represented the majority of NEPHRIN protein on the cell surface^17,19^, the R460Q mutant was likely to be localized on the cell surface of HEK293 cells. Thus, the underlying mechanisms for the pathogenesis may differ between Patient 3 (R460Q) and Patient 0 (E725D).

### Establishment of iPSCs from the two patients with *NPHS1* mutations

**Figure 3:**
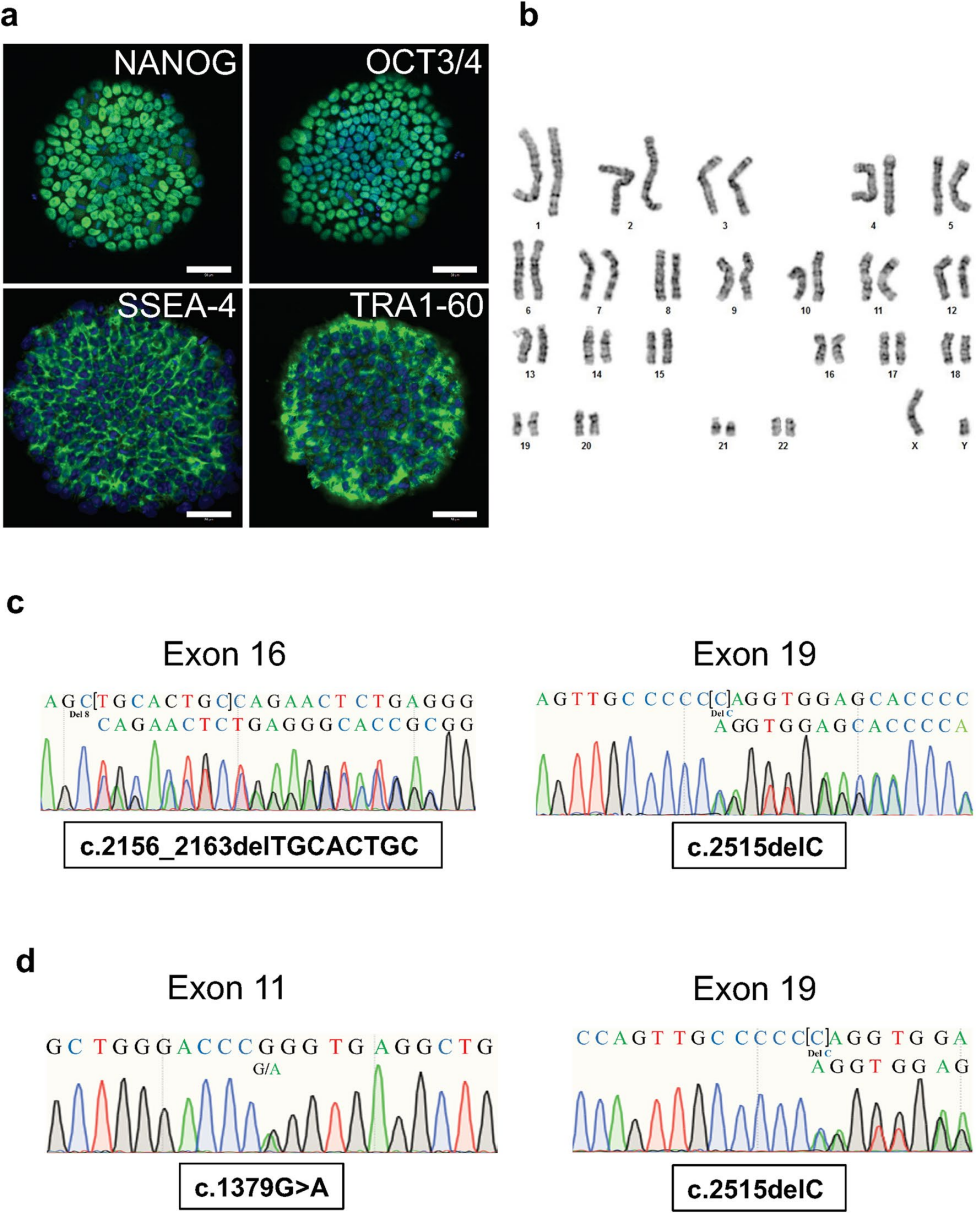
Establishment of iPSCs from the two patients with *NPHS1* mutations. (**a**) Stem cell markers expressed in the patient-derived iPSCs. Representative data for clone Pt3-32 are shown. The other three clones showed comparable results. Scale bars: 50 µm. (**b**) Normal karyotype (46, XY) in the patient-derived iPSCs (Pt-3–32). (**c**) *NPHS1* sequences in Patient 1-derived iPSCs. (**d**) *NPHS1* sequences in Patient 3-derived iPSCs.

It remained unclear whether the above findings held true in podocytes. Thus, we introduced reprogramming transcription factors into peripheral mononuclear cells from Patient 1 and Patient 3 using a Sendai virus vector, and established iPSC lines from both patients. The resulting cell lines expressed characteristic stem cell markers and had normal karyotypes (46, XY) (Fig. 3a,b). The patient-specific *NPHS1* mutations on both alleles were preserved in the iPSCs (Fig. 3c,d).

### NEPHRIN expression/localization is impaired in patient-derived podocytes

**Figure 4:**
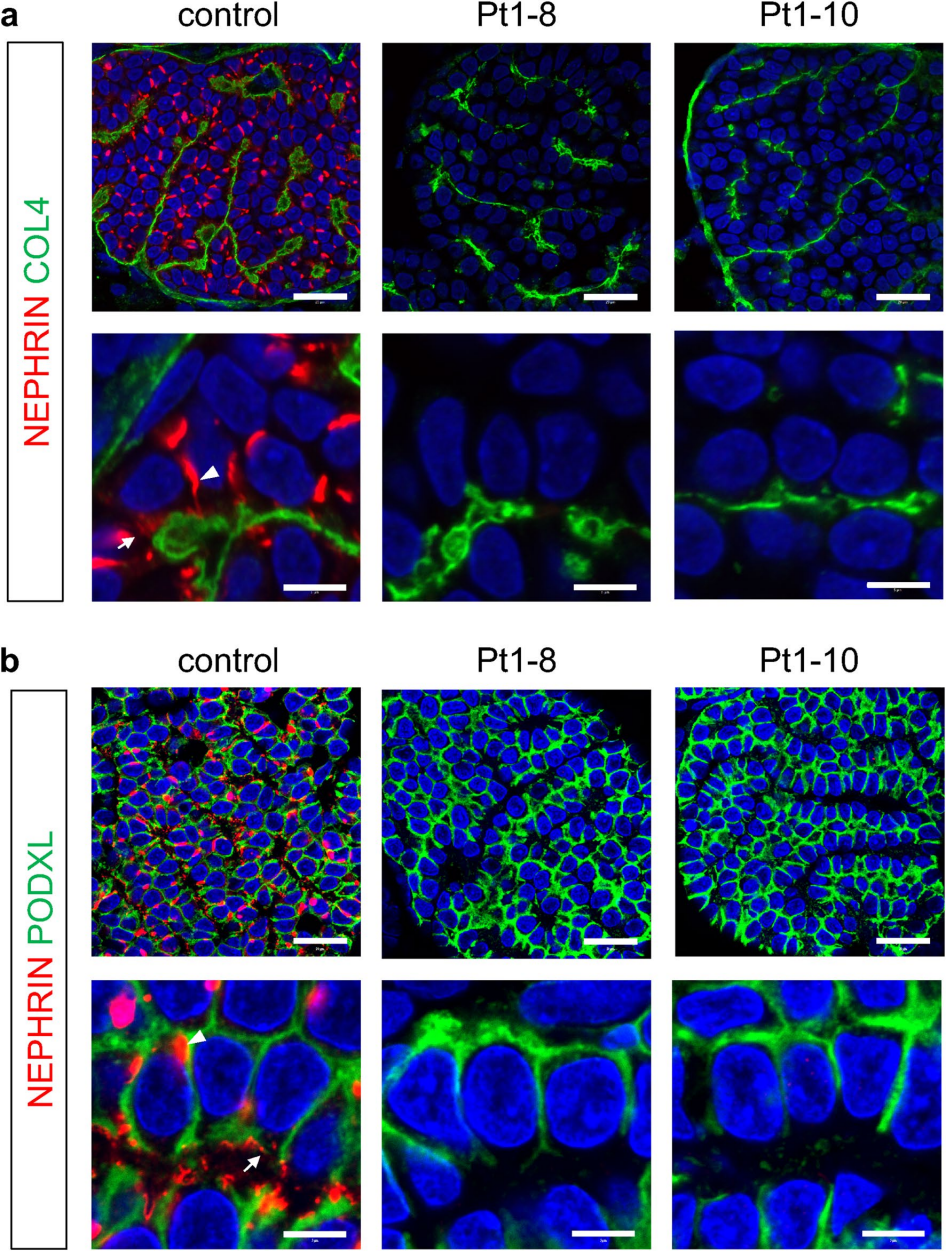
NEPHRIN expression is absent in Patient 1-derived podocytes. Section immunostaining of kidney organoids, showing the absence of NEPHRIN in Patient 1-derived podocytes (clones: Pt1-8, Pt1-10). Co-staining of NEPHRIN with type IV collagen (COL4) (**a**) or PODOCALYXIN (PODXL) (**b**) is shown. Scale bars: 20 µm for upper panels; 5 µm for lower panels.

**Figure 5:**
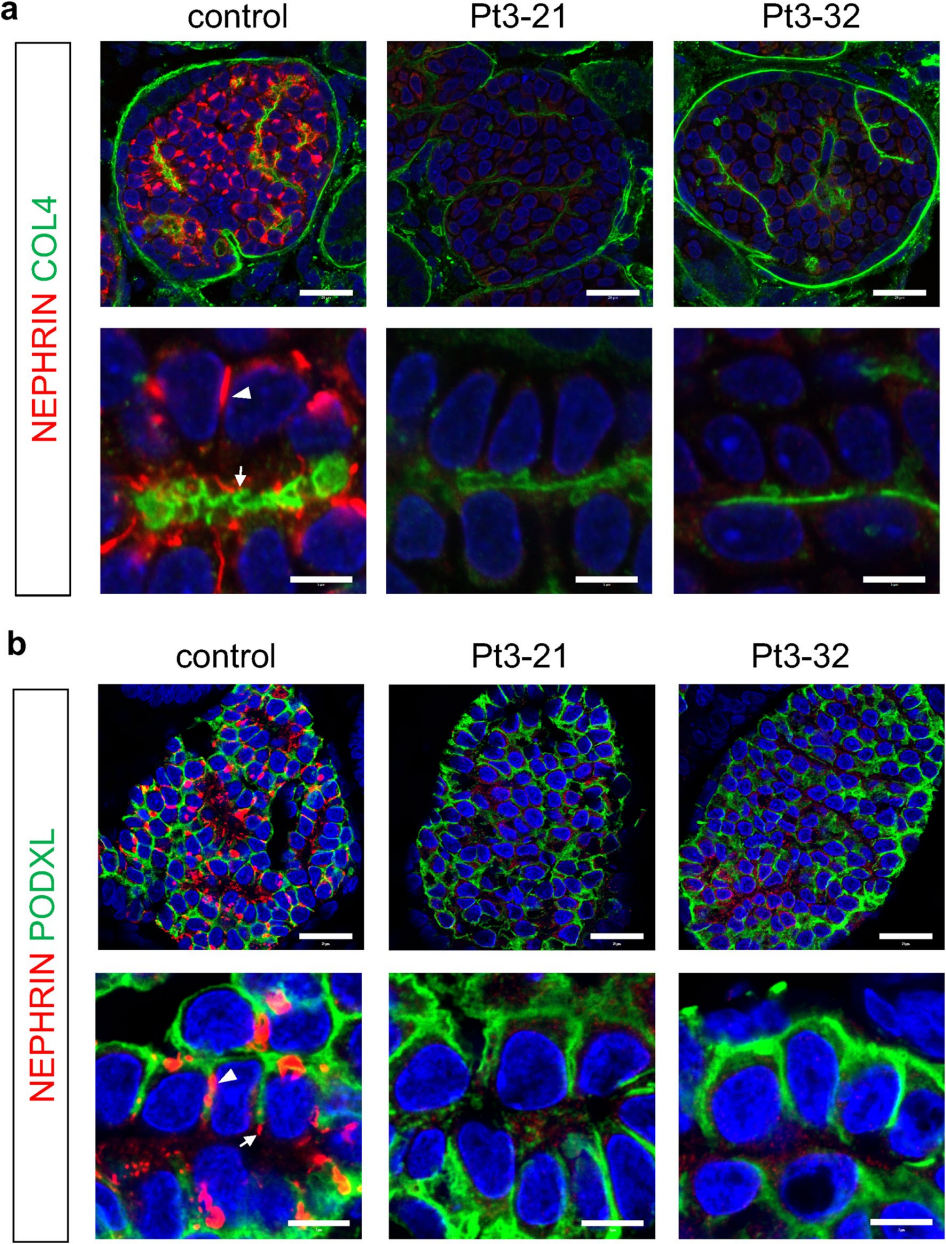
NEPHRIN localization is impaired in Patient 3-derived podocytes. Section immunostaining of kidney organoids, showing a reduction in NEPHRIN in Patient 3-derived podocytes (clones: Pt3-21, Pt3-32). Co-staining of NEPHRIN with type IV collagen (COL4) (**a**) or PODOCALYXIN (PODXL) (**b**) is shown. NEPHRIN is weakly detected in the cytoplasm. Arrowhead: lateral SD precursors; arrow: basal SD precursors. Scale bars: 20 µm for upper panels; 5 µm for lower panels.

We generated kidney organoids from the patient-derived iPSCs, based on our published protocol^16,20^. Both the control and mutant clones successfully formed kidney tissues containing glomeruli with podocytes (Figs. 4, 5). These podocytes were well equipped with apicobasal polarity, expressing comparable levels of the apical marker PODOCALYXIN (PODXL) and the basal marker type IV collagen (COL4). In the control organoids, we observed prominent NEPHRIN+ dot-like structures on the lateral side of most podocytes (Fig. 4a). NEPHRIN was also detected as smaller dots on the basal side adjacent to COL4+ basement membrane. These NEPHRIN+ domains did not overlap with PODXL+ apical domains (Fig. 4b). As we reported previously^17^, these dot-like structures likely reflect a transit state of NEPHRIN shifting from the lateral to basal domain of the podocytes, because NEPHRIN finally exhibited a basal localization upon organoid transplantation. Thus, we refer to these dot-like structures as SD precursors. Indeed, these structures were also observed in embryonic human podocytes in vivo^9^. In contrast, NEPHRIN or SD precursors were undetectable in the kidney organoids from Patient 1 with biallelic truncating mutations, while COL4 and PODXL expression remained unaffected (Fig. 4a,b). Unexpectedly, NEPHRIN was also markedly reduced in the organoids from Patient 3 with a missense mutation (R460Q). Specifically, it was weakly detected in the cytoplasm and SD precursors were rarely observed in the mutant podocytes (Fig. 5a,b). COL4 and PODXL expression remained unaffected (Fig. 5a,b). These features resembled those of the podocytes in organoids derived from Patient 0 (E725D) in our previous study^17^. Thus, in contrast to our observations in HEK293 cells and the previous report^7^, the R460Q mutant NEPHRIN protein failed to localize on the podocyte surface to form SD precursors.

**Figure 6:**
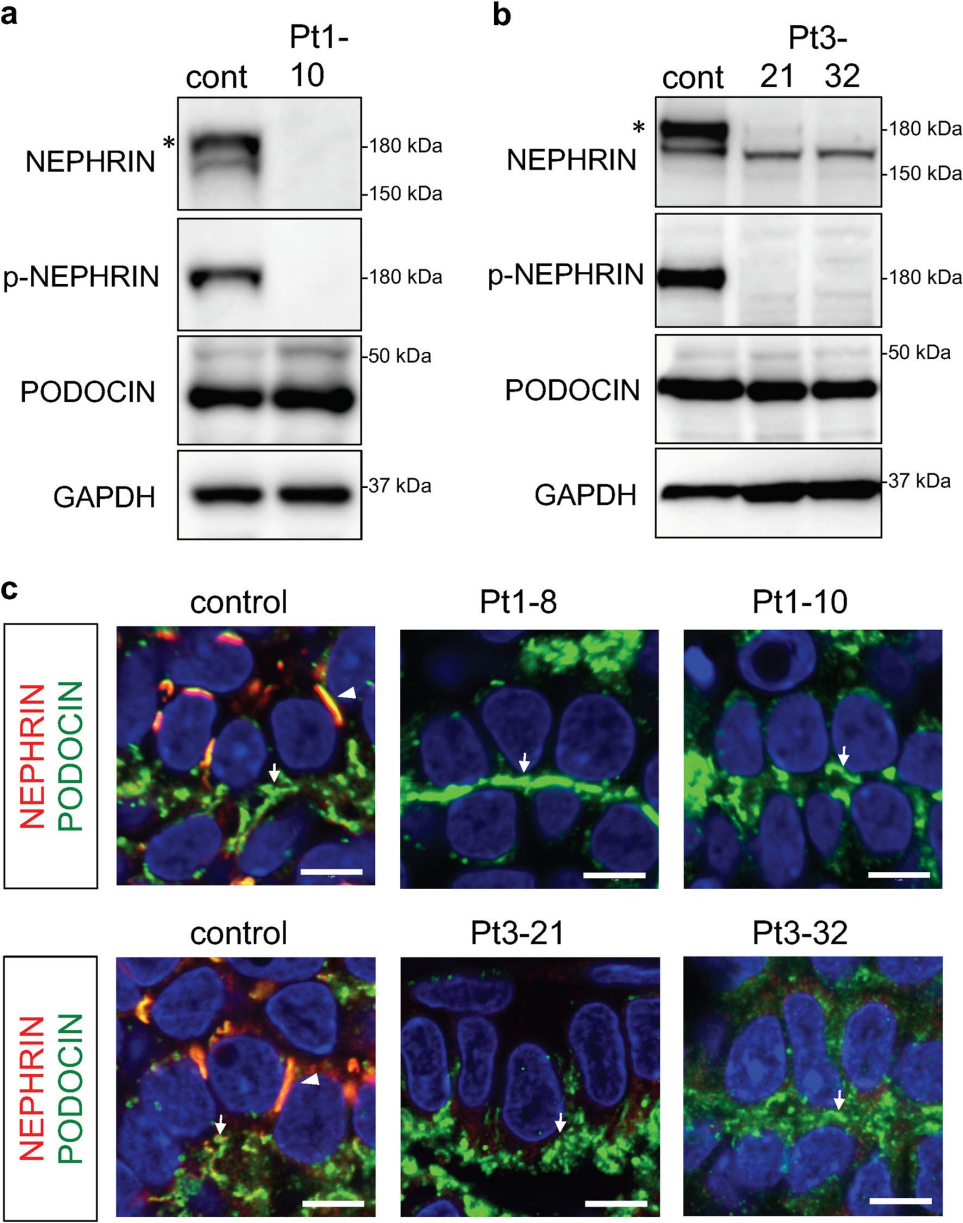
Localizations of NEPHRIN and PODOCIN are impaired in patient-derived podocytes. (**a**) Western blot analyses of NEPHRIN and PODOCIN in kidney organoids derived from Patient 1. NEPHRIN is absent in the patient-derived organoids. (**b**) Western blot analyses of NEPHRIN and PODOCIN in kidney organoids derived from Patient 3. The upper band of NEPHRIN (*) is missing in the patient-derived organoids. NEPHRIN phosphorylation is also undetectable. p-NEPHRIN: phosphorylated NEPHRIN. The results from the different blots were combined, as depicted by the solid boxes. (**c**) Section immunostaining of kidney organoids, showing the impaired localizations of NEPHRIN and PODOCIN in the patient-derived podocytes (upper row: Patient 1; lower row: Patient 3). PODOCIN is undetectable in the lateral domains of the podocytes derived from both patients. Arrowheads: lateral SD precursors; arrows: basal PODOCIN + domains. Scale bars: 5 µm.

Western blotting analysis showed that NEPHRIN expression disappeared in the kidney organoids derived from Patient 1 (Fig. 6a, Supplementary Fig. S2), confirming that the truncating mutations resulted in complete loss of the functional protein. NEPHRIN expression in Patient 3-derived organoids was also markedly affected (Fig. 6b, Supplementary Fig. S2). The upper band of NEPHRIN, which was predominant in the control organoids, was undetectable in the mutant organoids in sharp contrast to the observations in HEK293 cells. In previous studies, biotin labeling experiments in cell lines and isolated glomeruli in vitro, as well as in vivo labeling, demonstrated that the upper band corresponded to NEPHRIN protein on the cell surface^21–23^. Taken together with our immunostaining results showing impaired cell surface localization of the mutant proteins, the upper band in the organoids likely represented NEPHRIN protein on the cell surface, which was undetectable in the patient organoids. Indeed, NEPHRIN was not phosphorylated in the patient-derived organoids, but was phosphorylated in the control organoids (Fig. 6a,b), consistent with previous reports that tyrosine residues in NEPHRIN on the cell surface are phosphorylated^24,25^. Furthermore, these features in the western blot analyses resembled those in the organoids derived from Patient 0 (E725D) in our previous study^17^. Therefore, NEPHRIN localization is cell context-dependent, and kidney organoids likely represent the disease state more accurately than heterologous cells: the R460Q mutation impairs NEPHRIN localization on the podocyte surface.

### Localization of PODOCIN is impaired in both types of mutant podocytes

Western blotting analysis showed that the expression of another SD-associated protein, PODOCIN, was relatively unaffected in both types of mutant organoids (Fig. 6a,b, Supplementary Fig. S2). Histologically, PODOCIN was colocalized with NEPHRIN on the lateral and basal regions of the control podocytes (Fig. 6c, Supplementary Fig. S3). In contrast, PODOCIN was undetectable on the lateral SD precursor domains of the podocytes with the truncating mutations or R460Q missense mutation (Fig. 6c, Supplementary Fig. S3), indicating that lateral SD formation was dependent on NEPHRIN irrespective of the mutation types. However, PODOCIN was still observed on the basal side in both mutant podocytes (Fig. 6c, Supplementary Fig. S3). Thus, the overall basal distribution of PODOCIN was not affected by NEPHRIN absence or mislocalization, but NEPHRIN was required for recruitment of PODOCIN to the lateral SD precursor domains. These observations are consistent with those for the E725D mutation^17^, further supporting the notion that a similar mechanism may be operating for the pathogenesis: impaired NEPHRIN localization on the podocyte surface leading to failure of PODOCIN recruitment and SD precursor formation.

## Discussion

In the present study, we established iPSCs from two patients with different types of *NPHS1* mutations (truncating mutations and R460Q missense mutation) and examined the podocyte abnormalities in kidney organoids derived from these cells. NEPHRIN protein and SD precursors were undetectable in the former, as expected. While the latter missense mutation did not impair NEPHRIN localization in HEK293 cells, the mutant NEPHRIN in the organoids unexpectedly failed to accumulate on the podocyte surface to form SD precursors, indicating that NEPHRIN localization is cell context-dependent and that kidney organoids likely represent the disease state more accurately than heterologous cells: the R460Q mutation impairs NEPHRIN localization on the podocyte surface.

The observation of cell context-dependent NEPHRIN expression may be related to the protein degradation machinery. Misfolded mutant proteins retained in ER are usually degraded by the ERAD mechanism, and this machinery may be stricter in podocytes than in HEK293 cells. Alternatively, we overexpressed NEPHRIN in huge amounts in HEK293 cells using the potent cytomegalovirus promoter, and this may have allowed some of the mutant protein to escape the degradation machinery. Although the detailed mechanisms remain to be elucidated, the accumulated observations for normal cell surface localization of mutant NEPHRIN proteins in heterologous cells should be carefully interpreted and eventually tested using the kidney organoid platform, which can directly assess endogenous NEPHRIN. Having said that, it is still theoretically possible that the NEPHRIN protein from Patient 3 contained mutations other than R460Q that affected its localization. Although we did not detect any other pathogenic sequences in Patient 3, genetic correction of the R460Q mutation will unequivocally prove the role of this mutation.

Thus far, we have examined iPSCs from two patients with missense mutations (E725D and R460Q), both of which resulted in NEPHRIN mislocalization in podocytes. While it is necessary to search for patients with mislocalization-independent mechanisms, it currently seems practical to focus on NEPHRIN mislocalization and search for drugs that can relocate mutant full-length proteins to the correct positions. We recently developed a selective podocyte induction protocol from human iPSCs^26^, as well as a protocol for expansion of iPSC-derived nephron progenitors^27^. Application of these protocols to patient iPSCs will accelerate the screening efficiency for curative drugs. Because the responsiveness of organoids to individual compounds can be dependent on the mutations and genetic background, establishment of iPSC lines from multiple patients with missense mutations would be beneficial. Furthermore, the usefulness of these cell lines would not be limited to a specific congenital nephrotic syndrome. Many devastating kidney diseases in both children and adults start with proteinuria, and NEPHRIN localization and SD formation are affected in these diseases. If mechanisms to restore NEPHRIN localization can be identified, they could be applied to the development of novel treatments that can cure proteinuria and eventually renal failure.

iPSCs with truncating mutations may not be suitable for drug testing, because no full-length proteins are produced in the induced podocytes. Instead, complete lack of NEPHRIN will serve as an optimal negative control when searching for NEPHRIN complexes or downstream signaling events in kidney organoids. For example, kidney organoids from control and mutant iPSCs can be used for immunoprecipitation with anti-NEPHRIN antibodies, followed by mass spectrometry. Furthermore, the retention of PODOCIN on the basal side of podocytes even in the absence of NEPHRIN (see Fig. 6) may lead to the identification of NEPHRIN-independent complexes in human podocytes.

Taken together, we have established iPSCs from patients with truncating and missense mutations, and showed impairment of NEPHRIN localization in podocytes derived from these cells. Our established iPSCs and induction protocol will serve as valuable bases for dissecting the mechanisms underlying glomerular diseases in humans and for drug development toward specific therapies. To assist in achieving these goals, we have deposited all four iPSC lines (two from Patient 1 and two from Patient 3) in the RIKEN Bioresource Center, and have made them available to the research community.

## Methods

### Identification of *NPHS1* mutations in the patients and iPSCs

Genomic DNA was extracted from peripheral leukocytes in whole-blood samples using a DNA isolation kit (Takara). Individual exons of *NPHS1* were amplified by polymerase chain reaction (PCR). The primers for *NPHS1* were designed on the basis of previously published information regarding intron–exon boundaries^28^. The primer sequences used in this study were: EX10-11F, 5′-CACGATGGATAGGGGTGCTG-3′; EX10-11R, 5′-CCTGGTCCTTCCCCCACATT-3′; EX15-16F, 5′-CCTGATCTCCAATCTGTCCTTG-3′; EX15-16R, 5′-CCACAATGGGCAAGGTTCCTTG-3′; EX18-19F, 5′-GAGGCTACAGAAGGGACAATTTG-3′; and EX18-19R, 5′-GCTGGAGGTCCAGACCTGGG-3′. The PCR products were purified and sequenced. For sequencing of *NPHS1* in the iPSC clones, nested PCR was further performed to enrich specific fragments using the following primers: EX10-11 nestF, 5′-CCACGTCTGAAGCTCACTCC-3′; EX10-11 nestR, 5′-GTCCTTCCCCCACATTCCT-3′; Ex15-16 nestF: 5′-TGAAGACCGTCCAGAGTTCC-3′; Ex15-16 nestR, 5′-GTTCCTTGGGTGGGTGTG-3′; EX18-19 nestF, 5′-GGGACAATTTGGGCAGTGAT-3′; and EX18-19 nestR, 5′-CTCACCTGGGATCTTGGAGA-3′. All four iPSC clones showed identical mutations to those in the patients.

### Ethics status

All experiments using human samples were performed in accordance with institutional guidelines and approved by the licensing committees at the Faculty of Life Science, Kumamoto University: Ethics Committee for Epidemiological and General Research (approval number: 1453) and Ethics Committee for Human Genome and Gene Analysis Research (approval number: 359). After explaining our study, the parents of the patients agreed to participate in the study and signed informed consent forms.

### Generation of iPSCs from the patients

iPSCs were established using peripheral mononuclear cells obtained from the patients as described^29^. Briefly, peripheral mononuclear cells were stimulated with an anti-CD3 antibody (e-Bioscience; 16-0037-85) and introduced with four reprogramming transcription factors (OCT3/4, SOX2, KLF4, and MYC) using a Sendai virus vector (ID Pharma; CytoTune-iPS2.0). After establishment of the iPSC clones, the temperature-sensitive Sendai virus was eliminated by culture at 38 °C for 24 h. Among the resulting Sendai virus-free clones (10 clones from Patient 1 and 16 clones from Patient 3), two clones from Patient 1 (Pt1-8, Pt1-10) and three clones from Patient 3 (Pt3-8, Pt3-21, Pt3-32) were subjected to karyotyping, which confirmed that all five clones had normal karyotypes. Two clones from Patient 1 (Pt1-8, Pt1-10) and two clones from Patient 3 (Pt3-21, Pt3-32) were subsequently adapted to feeder-free conditions^30^ and used for further studies. Stem cell marker staining and karyotyping of these clones were performed as described^17^. All four clones were deposited in the RIKEN Bioresource Center (https://cell.brc.riken.jp/en/hps). Wild-type 201B7 cells were used as the control clone^31^.

### Kidney organoid induction from the patient-derived iPSCs

The patient-derived iPSC clones were induced toward nephron progenitors by a 13-day protocol as described^20,32^, and successful formation of the ITGA8+/PDGFRA− nephron progenitor fraction was confirmed by flow cytometry^32,33^, as shown in Supplementary Fig. S4. The nephron progenitor aggregates were induced with mouse embryonic spinal cord taken from E12.5 embryos as described^34^, and cultured at the air–fluid interface for 20 days on a polycarbonate filter (0.8 µm; Whatman) supplied with DMEM containing 10% KnockOut Serum Replacement (Thermo Fisher Scientific #10828028). All animal experiments were performed in accordance with institutional guidelines and approved by the Licensing Committee of Kumamoto University (#A2019-113). Two independent induction experiments were performed for each clone, and at least 16 organoids per clone were generated in each experiment. After confirming successful tubulogenesis under a stereomicroscope, three organoids per clone in each experiment were subjected to serial sectioning for immunostaining. 10–20 organoids were used for western blot analyses. Histological and western blotting analyses were performed as described previously^17^. The full-length western blots are shown in the Supplementary Figures. Information on the antibodies used was provided in the previous report^17^.

### Generation of HEK293 cell lines expressing mutant NEPHRIN proteins

Flp recombinase-mediated site-directed integration of exogenous NEPHRIN was performed as described^17^. Briefly, each mutant *NPHS1* gene was cloned into the pcDNA5/FRT/TO vector and transfected into Flp-In T-REx 293 cells with the pOG44 vector (Thermo Fisher Scientific), in accordance with the manufacturer’s instructions. Staining with or without detergent was carried out as described previously^17^. Cells were stained with either an antibody against the extracellular domain of NEPHRIN (50A9)^35^ (a kind gift from Dr. Karl Tryggvason, Karolinska Institute) in the absence of detergent or an antibody against the intracellular domain of NEPHRIN (Progen; GP-N2) in the presence of detergent (0.1% Triton X-100).

## Supplementary Information

Supplementary Information.

## Data Availability

No datasets were generated or analyzed during the current study.
