# Supplementary material for: Impaired NEPHRIN localization in kidney organoids derived from nephrotic patient iPS cells
Source: Sci Rep. 2021 Feb 17;11:3982. doi: 10.1038/s41598-021-83501-9 (PMC7890052; doi:10.1038/s41598-021-83501-9)
Supplement: Supplementary file 1 — Supplementary Information. [file 41598_2021_83501_MOESM1_ESM.pdf]

Supplementary information

Impaired NEPHRIN localization in kidney organoids derived from nephrotic patient iPS cells

Tomoko Ohmori[1], Shankhajit De[1], Shunsuke Tanigawa[1], Koichiro Miike[1], Mazharul Islam[1], Minami Soga[2], Takumi Era[2], Shinichi Shiona[3], Koichi Nakanishi[4,5], Hitoshi Nakazato[6], and Ryuichi Nishinakamura[1,*]

[1]Department of Kidney Development, Institute of Molecular Embryology and Genetics, Kumamoto University, Kumamoto 860-0811, Japan

[2]Department of Cell Modulation, Institute of Molecular Embryology and Genetics, Kumamoto University, Kumamoto 860-0811, Japan

[3]Department of Pediatrics, Oita Prefectural Hospital, Oita 870-0855, Japan

[4]Department of Child Health and Welfare (Pediatrics), Graduate School of Medicine, University of the Ryukyus, Okinawa 903-0215, Japan

[5]Genetic Consulting Unit, University of the Ryukyus Hospital, Okinawa 903-0215, Japan

[6]Department of Pediatrics, Faculty of Life Sciences, Kumamoto University, Kumamoto 860-8556, Japan

Supplementary Figure S1

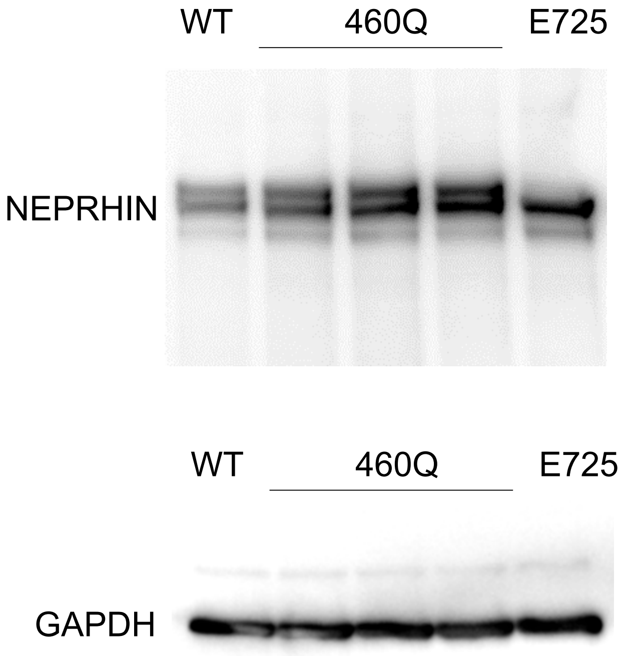

Supplementary Figure S1

The full-length western blots used for Figure 2

Western blotting analysis of HEK293 cells overexpressing wild-type (WT), R460Q mutant (Patient 3), and E725D mutant (Patient 0) NEPHRIN. GAPDH is used as a loading control.

Supplementary Figure S2

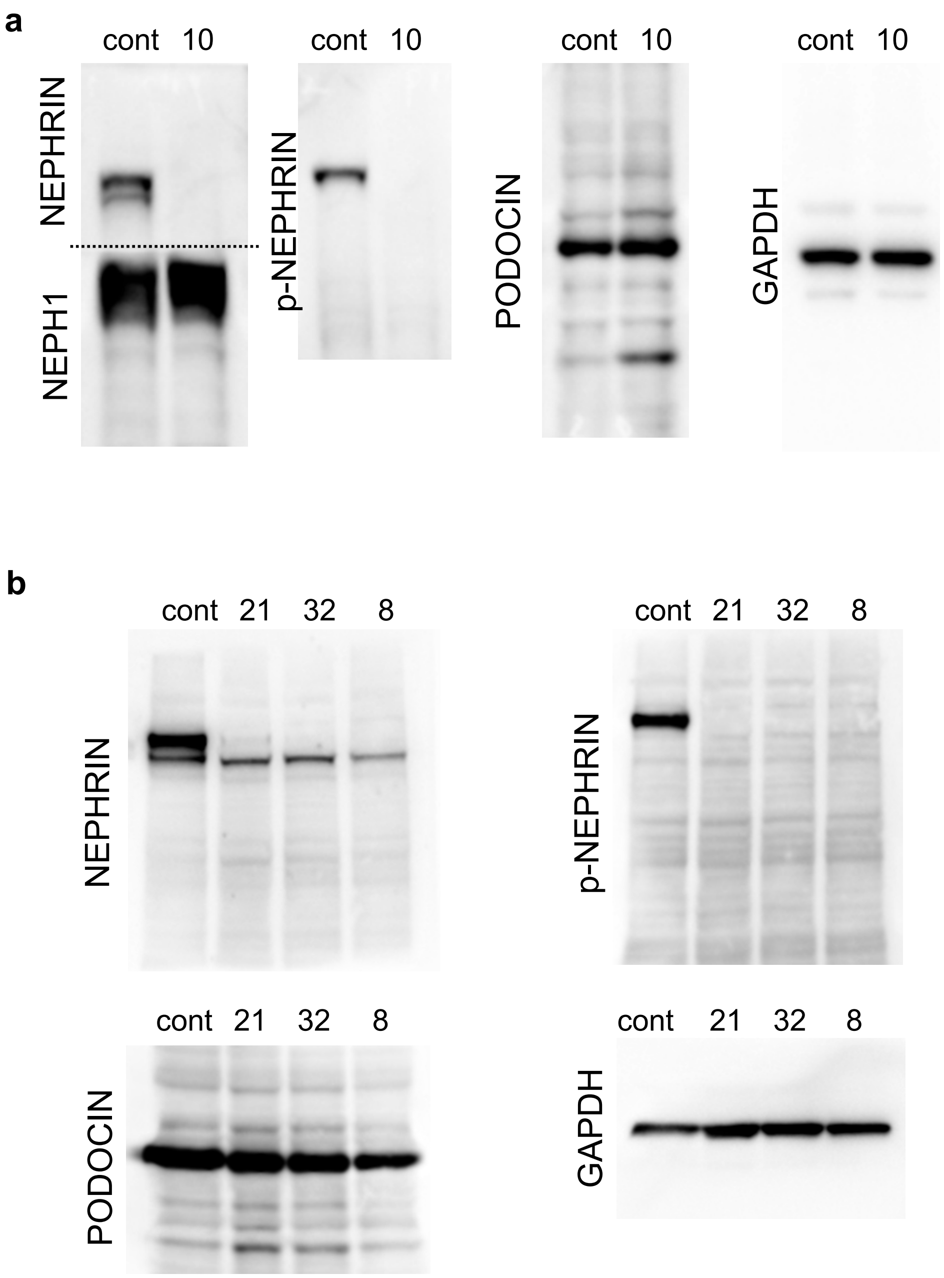

Supplementary Figure S2

The full-length western blots used for Figure 6

Western blot analyses of NEPHRIN, phosphorylated NEPHRIN (p-NEPHRIN), and PODOCIN in kidney organoids derived from Patient 1 **(a)** and 3 **(b)**. The clone numbers are indicated at the top. GAPDH is used as a loading control. cont: control clones.

Supplementary Figure S3

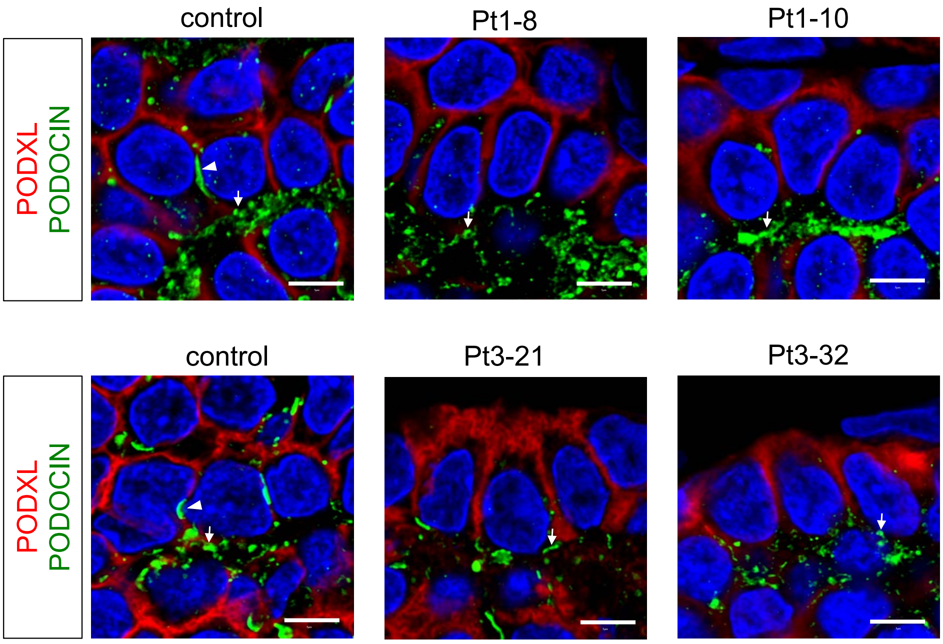

Supplementary Figure S3

Localization of PODOCIN is impaired in patient-derived podocytes

Section immunostaining of kidney organoids was performed with anti-PODOCIN and anti-PODXL antibodies (upper row: Patient 1; lower row: Patient 3). PODOCIN is undetectable in the lateral domains of the podocytes derived from both patients.

Arrowheads: lateral SD precursors; arrows: basal PODOCIN+ domains. Scale bars: 5 μm.

Supplementary Figure S4

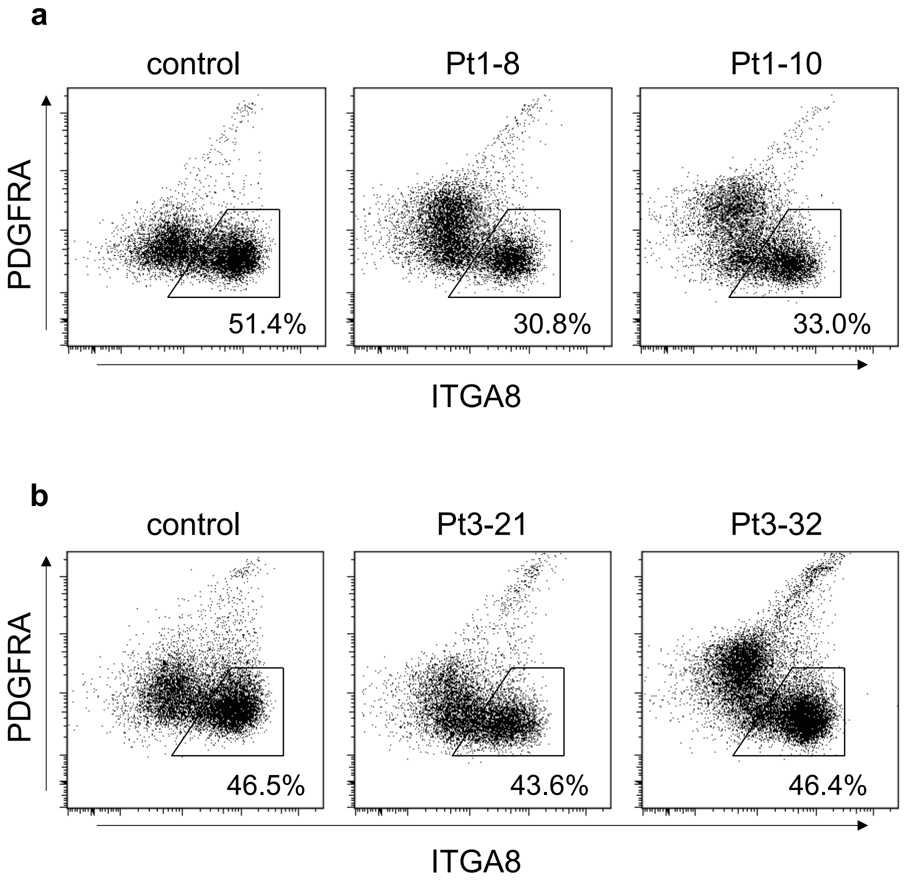

Supplementary Figure S4

Induction of nephron progenitors from iPSCs

Induction was performed for the control clone (201B7), two Patient 1-derived clones (Pt1-8, Pt1-10), and two Patient 3-derived clones (Pt3-21, Pt3-32), and the formation of ITGA8+/PDGFRA− nephron progenitors was analyzed by flow cytometry on day 13. Representative data are shown.
